# RETRACTION: Dentatin Induces Apoptosis in Prostate Cancer Cells via Bcl‐2, Bcl‐xL, Survivin Downregulation, Caspase‐9, ‐3/7 Activation, and NF‐κB Inhibition

**DOI:** 10.1155/ecam/9806757

**Published:** 2026-05-07

**Authors:** Evidence-Based Complementary and Alternative Medicine

RETRACTION: I. A. Arbab, C. Y. Looi, A. B. Abdul, et al., “Dentatin Induces Apoptosis in Prostate Cancer Cells via Bcl‐2, Bcl‐xL, Survivin Downregulation, Caspase‐9, ‐3/7 Activation, and NF‐κB Inhibition,” *Evidence-Based Complementary and Alternative Medicine* 2012, no. 1 (2012): 856029, https://doi.org/10.1155/2012/856029.

The above article, published online on 03 October 2012 in Wiley Online Library (https://wileyonlinelibrary.com), has been retracted by John Wiley & Sons Ltd. The retraction has been agreed due to the following figure concerns:

• Figure 11, panel “Vehicle CMC‐Liver” shares unexpected similarities with Figure 4, panel “B2” in [1] and Figure 2e in [2];
• Figure 11, panel “Dentatin 100 mg/kg‐Liver” shares unexpected similarities with Figure 2a in [3] and Figure 1b in [4];
• Figure 11, panel “Dentatin 1000 mg/kg‐Liver” shares unexpected similarities with Figure 10e in [5].

Several attempts by the journal to contact the authors did not result in a response. As such, the results and conclusions cannot be considered as reliable.

## References

[1] Wong W.-L. , Abdulla M. A. , Chua K.-H. , Kuppusamy U. R. , Tan Y.-S. , and Sabaratnam V. , Hepatoprotective Effects of *Panus giganteus* (Berk.) Corner Against Thioacetamide- (TAA-) Induced Liver Injury in Rats, Evidence-Based Complementary and Alternative Medicine. (2012) 2012, no. 1, 10.1155/2012/170303, 2-s2.0-84862289223. PMC3357533 22649470

[2] Salama S. M. , Abdulla M. A. , AlRashdi A. S. , Ismail S. , Alkiyumi S. S. , and Golbabapour S. , Hepatoprotective Effect of Ethanolic Extract of *Curcuma longa* on Thioacetamide Induced Liver Cirrhosis in Rats, BMC Complementary and Alternative Medicine. (March 2013) 13, no. 1, 10.1186/1472-6882-13-56, 2-s2.0-84874467558. PMC3605171 23496995

[3] Alnajar Z. A. A. , Abdulla M. A. , Ali H. M. et al., Acute Toxicity Evaluation, Antibacterial, Antioxidant and Immunomodulatory Effects of *Melastoma malabathricum* , Molecules. (March 2012) 17, no. 3, 3547–3559, 10.3390/molecules17033547, 2-s2.0-84858990466. 22433579 PMC6268612

[4] Ibrahim I. A. A. , Qader S. W. , Abdulla M. A. et al., Effects of *Pithecellobium jiringa* Ethanol Extract Against Ethanol-Induced Gastric Mucosal Injuries in Sprague-Dawley Rats, Molecules. (March 2012) 17, no. 3, 2796–2811, 10.3390/molecules17032796, 2-s2.0-84858963805. 22395408 PMC6268751

[5] Amin Z. A. , Ali H. M. , Alshawsh M. A. , Darvish P. H. , and Abdulla M. A. , Application of *Antrodia camphorata* Promotes Rat’s Wound Healing *In Vivo* and Facilitates Fibroblast Cell Proliferation *In Vitro* , Evidence-Based Complementary and Alternative Medicine. (2015) 2015, no. 1, 10.1155/2015/317693, 2-s2.0-84945296345. PMC4617886 26557855

